# A Comparative Evaluation of Subjective Patient Comfort Levels Between Non-latex Conventional Dental Dam System and Prefabricated Non-latex Dental Dam System: A Prospective Interventional Study

**DOI:** 10.7759/cureus.112132

**Published:** 2026-07-06

**Authors:** Shagun Gill, Aakash Gupta, Angad Gill, Aditi Kulshrestha, Tansha, Somya Chhabra

**Affiliations:** 1 Department of Conservative Dentistry and Endodontics, Gian Sagar Dental College and Hospital, Rajpura, IND; 2 Department of Dentistry, All India Institute of Medical Sciences, Bathinda, Bathinda, IND; 3 Department of Transfusion Medicine, Government Medical College and Hospital, Chandigarh, IND; 4 Department of Conservative Dentistry and Endodontics, Manav Rachna Dental College, Faridabad, IND; 5 Department of Conservative Dentistry and Endodontics, Maharishi Markandeshwar College of Dental Sciences and Research, Ambala, IND; 6 Department of General Dentistry, Family Dental Clinic, New Delhi, IND

**Keywords:** aseptic environment, cotton rolls, dental isolation, flexidam, isolite, latex allergy, optidam, optradam, patient comfort, rubber dam

## Abstract

Background

Dental dam isolation is considered the standard of care in endodontics due to its role in maintaining an aseptic operating field and enhancing patient safety. However, limited evidence exists comparing patient comfort with non-latex conventional and prefabricated dental dam systems.

Objectives

The primary objective of this randomised interventional questionnaire-based study was to evaluate and compare subjective patient comfort levels between a non-latex conventional and a non-latex prefabricated dental dam system during endodontic treatment of mandibular first molars.

Materials and methods

Sixty volunteers were randomly assigned to two equal groups (n = 30 each). After obtaining informed consent, each dental dam system was applied, and endodontic treatment was performed on mandibular first molars. Upon completion of the procedure, patients evaluated their comfort by filling out a custom-designed questionnaire. Data were analysed using IBM SPSS Statistics for Windows, Version 22.0 (Released 2013; IBM Corp., Armonk, NY, USA) utilising the Pearson’s Chi-square test for the qualitative variables.

Results

Conventional and prefabricated non-latex dental dam systems provided similar levels of patient comfort. Stretching sensation, difficulty in swallowing, unpleasant taste or odour, nausea, and overall patient experience did not differ significantly (p > 0.05), with the majority of patients expressing a positive treatment experience.

Conclusion

Patients experienced similar comfort while undergoing treatment with non-latex conventional and prefabricated dental dam systems.

## Introduction

Since its creation by S. C. Barnum in 1864, the "Rubber Dam" (RD) has revolutionised dentistry to the extent that it is now recognised as the "Standard of Care," particularly in endodontics [1]. The COVID-19 pandemic further emphasised its importance as an effective barrier against aerosol transmission. National and international guidelines by the American Dental Association (ADA), World Health Organization (WHO), and Centers for Disease Control and Prevention (CDC) strongly recommend its use during dental restorative and endodontic procedures [2,3].

Quantitative evidence strongly supports these recommendations. Studies have demonstrated that dental dam isolation can reduce bacterial aerosol contamination by up to 98.8% at one meter from the operative site, with an even greater reduction of up to 99.4% when combined with pre-procedural antiseptic mouth rinses. It also significantly lowers ultrafine particle counts in dental aerosols when used with high-volume suction, thereby reducing cross-contamination [4]. Besides providing an aseptic and dry operating site, a dental dam increases operator efficiency by increasing visibility, facilitating soft tissue retraction, reducing rinsing, and decreasing patient interactions [5]. It also reduces the risk of aspiration and swallowing. It protects the patient's soft tissues from irritant dental materials [6]. The dental dam also improves the physical and chemical properties of dental materials by reducing moisture contamination of the target site from oral fluids during the insertion of restorative materials, thereby strengthening their chemical bonding to enamel and dentin [7].

Despite these benefits, traditional dental dam setups have drawbacks, such as the latex material used for their fabrication, which might serve as a possible allergen for certain patients, longer application time, frame-related pressure, difficulty in taking radiographs, and patient discomfort [8]. This led to the development of prefabricated dental dam systems designed to overcome these limitations.

However, comparative data on non-latex variants, which are critical for patients with latex allergy, remain limited. This study addresses this gap by evaluating subjective patient comfort with non-latex conventional and prefabricated systems.

## Materials and methods

Study design and setting

A single-centre, prospective, interventional study was conducted at Baba Jaswant Singh Dental College, Hospital and Research Institute (BJSDC), Ludhiana, affiliated with Baba Farid University of Health Sciences (BFUHS), Faridkot, India.

Several operators carried out this investigation. For each patient, all the procedures were carried out by a single clinician. The institute's Department of Conservative Dentistry and Endodontics conducted a pilot study of the questionnaire. A computer-generated randomisation sequence was used to divide the participants into two groups in a 1:1 ratio. An investigator who was not involved in participant recruitment created the sequence. Sequentially numbered, opaque, sealed envelopes were used to preserve allocation concealment until assignment time.

Ethical clearance and approval certification

The protocol of this study was approved by Baba Farid University of Health Sciences (BFUHS), Faridkot, vide Approval Certification No. BFUHS/2k22/p-TH/12220, dated November 23, 2022. The Certificate of Clearance from the Institutional Ethical Committee was issued by the Chairman of the Institutional Ethical Committee of BJSDC, Ludhiana, on May 4, 2022.

Participants, study area, and study duration

Sixty patients aged between 18 and 44 years who reported to the Department of Conservative Dentistry and Endodontics at BJSDC from December 2022 to February 2024 and required endodontic treatment in the mandibular first molars were chosen for the study based on predetermined selection criteria.

Sample size calculation

The sample size was determined prior to the commencement of the study using the following formula: $N = \frac{2 \left( Z_{\alpha/2} \right)^2 s^2}{d^2}$, where $N$ denotes the required sample size per group, $s$ represents the standard deviation, $d$ is the minimum clinically significant difference, and $Z_{\alpha/2}$ is the standard normal deviate corresponding to a two-tailed significance level.

For the present study, the standard deviation was taken as $s = 2.25$, the minimum clinically significant difference as $d = 1.86$, and the level of significance was set at 5% ($\alpha = 0.05$), corresponding to a $Z_{\alpha/2}$ value of 1.96. The statistical power was fixed at 80%.

Based on these assumptions, the minimum sample size required was calculated to be 12 participants per group. To account for possible dropouts and improve the reliability of the study results, the sample size was increased to 30 participants in each group. Consequently, a total of 60 patients were included in the study and underwent dental dam application.

Inclusion and exclusion criteria

Patients were screened for respiratory disorders, including asthma, chronic obstructive pulmonary disease (COPD), and tuberculosis. Patients having latex allergy were included, while patients having any respiratory disorders or a habit of mouth breathing were excluded from the study. The selected patients were then randomly divided into two groups. The permanent mandibular first molars requiring endodontic treatment that were fully erupted, periodontally sound, and free of any crown or bridge restorations were included, whereas permanent mandibular first molars that were partially erupted, had subgingival/root caries, or were extremely malpositioned were excluded from the study.

Informed consent

All the patients were given a complete verbal and written explanation, in three different languages, of the procedure, the need for, and the safety of the RD application. An informed consent form and a medical history questionnaire were thereafter obtained.

Clinical procedure

**Figure 1:**
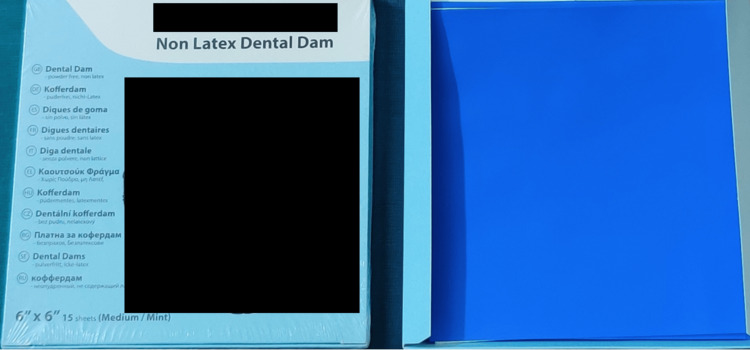
Conventional non-latex dental dam system (Group A).

**Figure 2:**
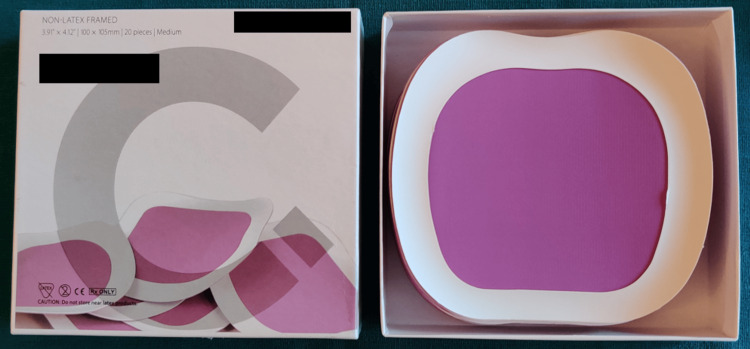
Prefabricated non-latex dental dam system (Group B).

Following the administration of local anaesthesia, patients from Group A (n = 30) underwent endodontic root canal treatment of a permanent mandibular first molar using a conventional non-latex dental dam system (Figure 1). In contrast, patients from Group B (n = 30) underwent treatment using a prefabricated non-latex dental dam system (Figure 2). To standardise the procedure, the universal mandibular molar clamp (#W56) was utilised across all subjects in both groups. To eliminate operator-induced bias, patients were left alone in the operatory and asked to complete a custom-designed questionnaire to summarise their overall experience.

Statistical analysis

For data collection, Microsoft Excel software (Microsoft® Corp., Redmond, WA, USA) was used. All collected data were analysed using IBM SPSS Statistics for Windows, Version 22.0 (Released 2013; IBM Corp., Armonk, NY, USA). The results were presented as mean ± standard deviation. The qualitative variables were compared using Pearson’s Chi-square test. A (probability value) p-value <0.05 was assumed to be statistically significant.

## Results

Most patients in Group A and Group B reported only mild stretching of the lips (24 (80%) and 27 (90%), respectively) and the tongue (28 (93.3%) and 29 (96.67%), respectively). The intergroup differences regarding soft tissue retraction were not statistically significant (Table 1).

**Table 1:** Intergroup comparison of subjective stretching sensations of the lips and tongue between patients treated with a conventional non-latex dental dam system (Group A) and a prefabricated non-latex dental dam system (Group B). Data are presented as frequency (n) and percentage (%). Total sample size = 60 (30 patients per group). p < 0.05 was considered to be statistically significant. χ² = chi-square statistic; df = degrees of freedom; n = number of patients, p-value = probability value; Group A = conventional non-latex dental dam system; Group B = prefabricated non-latex dental dam system.

Subjective Parameter	Group	Mild n (%)	Moderate n (%)	Severe n (%)	χ²	df	p-value	Significance
Stretching of Lips	Group A	24 (80)	6 (20)	0 (0)	0.741	1	0.389	Not significant
	Group B	27 (90)	3 (10)	0 (0)				
Stretching of the Tongue	Group A	28 (93.33)	2 (6.67)	0 (0)	0.351	1	0.554	Not significant
	Group B	29 (96.67)	1 (3.33)	0 (0)				

In Groups A and B, four (13.33%) and two (6.67%) of patients experienced difficulty in swallowing, respectively. In Groups A and B, none of the subjects experienced nausea or unpleasant odour. In Groups A and B, 7 (26.67%) and 11 (36.67%) patients experienced an unpleasant taste, respectively. The intergroup comparison between Groups A and B was not statistically significant (Table 2).

**Table 2:** Intergroup comparison of subjective parameters, including difficulty in swallowing, nausea, unpleasant odour, and unpleasant taste, between patients treated with a conventional non-latex dental dam system (Group A) and a prefabricated non-latex dental dam system (Group B). Data are presented as frequency (n) and percentage (%). Total sample size = 60 (30 patients per group). p < 0.05 was considered to be statistically significant. χ² = chi-square statistic; df = degrees of freedom; n = number of patients; NA = not applicable, p-value = probability value; Group A = conventional non-latex dental dam system; Group B = prefabricated non-latex dental dam system.

Subjective Parameter	Group	Yes n (%)	No n (%)	χ²	df	p-value	Significance
Difficulty in swallowing	Group A	4 (13.33)	26 (86.67)	0.741	1	0.389	Not significant
	Group B	2 (6.67)	28 (93.33)				
Nausea	Group A	0 (0)	30 (100)	NA	NA	>0.99	Not significant
	Group B						
Unpleasant odour	Group A	0 (0)	30 (100)	NA	NA	>0.99	Not significant
	Group B						
Unpleasant taste	Group A	7 (26.67)	23 (73.33)	1.271	1	0.259	Not significant
	Group B	11 (36.67)	19 (63.33)				

In Group A, 25 (83.33%) patients had a pleasant experience, while in Group B, 27 (90%) patients had a pleasant experience while undergoing treatment with the isolation system. The intergroup comparison between Groups A and B was not statistically significant (Table 3). This suggests that there was no difference in the patients’ comfort level between Groups A and B.

**Table 3:** Intergroup comparison of the overall patient experience with the isolation system used between patients treated with a conventional non-latex dental dam system (Group A) and a prefabricated non-latex dental dam system (Group B). Data are presented as frequency (n) and percentage (%). Total sample size = 60 (30 patients per group). p < 0.05 was considered to be statistically significant. χ² = chi-square statistic; df = degrees of freedom; n = number of patients, p-value = probability value; Group A = conventional non-latex dental dam system; Group B = prefabricated non-latex dental dam system.

Overall Experience	Group A n (%)	Group B n (%)	χ²	df	p-value	Significance
Pleasant	25 (83.33)	27 (90)	1.270	1	0.399	Not significant
Unpleasant	5 (16.67)	3 (10)				

## Discussion

The aim of this study was to compare the subjective patient comfort levels with a non-latex conventional and non-latex prefabricated dental dam system. The findings of this study suggest that both dental dam systems were equally comfortable in terms of their stretching sensation, difficulty in swallowing, nausea, unpleasant odour, and taste.

Certain chemicals and additives used in the production of latex have been linked to dermatitis and other skin and mucosal irritations. According to Salomez et al. [9], true latex allergies typically arise from an inflammatory reaction to naturally occurring proteins present in latex. *Hevea brasiliensis* (Hev b), a rubber tree, secretes an intracellular cytosol known as natural rubber latex (NRL), which serves as a protective sealant. Due to its exceptional qualities, it is a significant biopolymer that is used in numerous applications. The WHO/International Union of Immunologic Societies Allergen Nomenclature Committee has identified and classified 15 polypeptides (Hev b 1-15) as possible allergens. The current global average prevalence of latex allergy is 4.3% in the general population. According to Nucera et al. [10], the prevalence of latex allergy is 7.2%, and the prevalence of sensitisation among vulnerable people is 30.4%. Since it is not possible to determine whether a patient is allergic to latex before treatment, non-latex dental dam sheets are the safer and preferred option [11]. Since both sheets used in this study were non-latex, the patients did not experience any skin irritation due to the absence of potential allergenic peptides.

Organic compounds that gradually evaporate into the atmosphere are known as volatile organic compounds (VOCs). When rubber is exposed to the air, particularly in warm weather, these compounds, which are frequently retained within the rubber during manufacturing, are released. The main source of the rubber scent is this process, sometimes referred to as off-gassing or out-gassing. Rubber sheets can be made more flexible and durable by adding certain chemicals, such as naphthenic oils and non-staining antioxidants. However, particularly when they are fresh, these compounds may smell strongly. These additives are found in synthetic rubber, such as neoprene or nitrile, and they add to the unique scent. An early smell may also result from the vulcanisation process, which adds sulphur to rubber at high temperatures to harden it. Rubber becomes tougher and more resilient after vulcanisation, although the remaining sulphur compounds may still smell until they are completely gone [12]. Since the sheets used in this study were latex-free and made of a polyisoprene-based material, which has a polymer structure akin to natural rubber but without the potential allergens found in latex proteins [13], the patients did not experience any foul odour or nausea.

Similar results were obtained in a previously conducted in vivo study comparing a prefabricated (Isolite) dental dam system with a traditional dental dam system to investigate patient satisfaction with both isolation systems. The patients' subjective degree of comfort was measured using a visual analogue scale (VAS). It was discovered that the participants' preferences for both approaches were equal. This was attributed to reduced gagging, less fluid accumulation, and the built-in continuous suction that kept the mouth drier. The system's design also provided better illumination and cheek and tongue retraction without the full-enclosure feeling of a traditional dam, making it less claustrophobic for children [14]. The results obtained in this study also align with previously conducted comparative trials. Kapitán et al. [15] reported that 77% of patients experienced higher comfort with a dental dam, with 86% preferring it for future treatments. This was attributed to a lower risk of aspiration or ingestion of dental materials (71%), less water buildup in the oral cavity (68%), better overall treatment quality (68%), less chemical irritation of soft tissues (52%), and a lower risk of cross-infection (43%). Patients valued the drier field and efficient soft tissue retraction, which reduced disruptions and the feeling of "drowning" in water or saliva spray. A 2025 paediatric study found that RD isolation was not stressful for children compared with cotton rolls. Additionally, breathing comfort was highlighted with adjunct devices [16,17]. High comfort levels were linked to effective moisture control and soft tissue protection, which reduced procedural interruptions, material contamination concerns, and the need for frequent suctioning or rinsing.

While Binqali et al. [8] noted benefits for the prefabricated OptiDam in terms of ease of use and patient comfort, high comfort levels were observed with both systems. Feierabend et al. [6] reported higher patient and clinician acceptance of the conventional dental dam over a framed prefabricated system (Optradam). These discrepancies could be caused by changes in the framed system design (tension and stiffness), operator experience with conventional setups, particular product features, patient demographics, or study procedures. The comparable comfort levels seen in the current investigation were probably influenced by the use of contemporary non-latex systems in standardised adult mandibular molar endodontic cases.

The table below compares the present study with recent comparative trials on dental dam systems. Patient comfort remained high across both systems (typically 83%-97%) (Table 4).

**Table 4:** Comparative analysis of various studies conducted on dental dam systems comparing them in terms of patient satisfaction with the current study.

Study (Year)	Sample & Design	Systems Compared	Patient Comfort	Main Findings
Current Study: Gill et al. (2026)	60 adults (n=30/group), in vivo RCT, Mandibular molars	Conventional (non-latex) vs. Prefabricated (non-latex)	Similar (83.3% vs 90%; pleasant experience; not significant)	Comparable patient comfort
BaHammam et al. (2025) [18]	Preclinical simulation, Crossover (n=94 students)	Conventional vs. OptraDam Plus	Not the primary outcome	Conventional is better for isolation quality & feasibility in training
Binqali et al. (2025) [8]	RCT, Routine dental procedures	Conventional RD vs. OptiDam	High in both; OptiDam showed advantages in comfort	OptiDam advantages in ease & comfort; Both are effective
Waheed et al. (2024) [19]	Clinical trial (n=60)	Conventional vs. OptraDam Plus	Conventional: 96.7% comfortable OptraDam: 86.7%	Conventional preferred by patients (73.3%) & operators (80%)
Kapitán et al. (2015) [15]	In vivo clinical testing	Conventional vs. OptraDam Plus vs. OptiDam	77-86.7% higher comfort with RD vs. no RD; OptiDam ranked highest	OptiDam best overall balance; Good isolation across systems
Feierabend et al. (2011) [6]	Clinical comparison	Conventional vs. New framed systems (Optradam)	Better acceptance with conventional	Conventional preferred due to operator familiarity; newer designs don't necessarily imply better acceptance

Besides the parameters discussed in this study, other parameters, such as the price, operator's preference, colour, and thickness of the membranes, also play an important role. The cost-effectiveness of the conventional systems may influence the choice of the isolation system.

Limitations of this study include its single-centre design and focus on only mandibular first molars in only 60 adult patients aged between 18 and 44 years. Broad, multicentre trials with a large sample size that evaluate diverse teeth among various age groups would help to better generalise the outcomes. Operator experience, pre-procedure patient education, and patients with hypersalivation or xerostomia can also influence comfort metrics across studies.

## Conclusions

This study demonstrates that both non-latex conventional and prefabricated dental dam systems offer comparable levels of patient comfort during endodontic treatment. No statistically significant differences were found between the groups across key comfort parameters, including lip and tongue stretching, difficulty in swallowing, unpleasant taste or odour, nausea, and overall experience. Consequently, both systems can be confidently employed in daily clinical practice without compromising patient perception or satisfaction.

While prefabricated systems offer practical advantages, such as faster application and easier radiographic access, conventional systems remain a cost-effective option. Clinicians should therefore select the most appropriate system based on operator preference, case-specific needs, and economic considerations rather than patient comfort alone. Larger multicentre studies across diverse populations and tooth types are warranted to further validate these findings and enhance generalisability.
